# Contextual and experiential aspects of the psychedelic experience predicting improvement in subjective wellbeing: results from a Norwegian internet convenience sample

**DOI:** 10.3389/fphar.2025.1556299

**Published:** 2025-04-10

**Authors:** Paula Aarseth Tunstad, Tor-Morten Kvam, Malin V. Uthaug, Lowan H. Stewart, Kristoffer A. A. Andersen, Cato Grønnerød

**Affiliations:** ^1^ Indre Sogn Regional Psychiatric Clinic, Førde, Norway; ^2^ Faculty of Medicine, University of Oslo, Oslo, Norway; ^3^ Nordre Østfold DPS, Østfold Hospital Trust, Grålum, Norway; ^4^ Department of Neuropsychology and Psychopharmacology, Faculty of Psychology and Neuroscience, Maastricht University, Maastricht, Netherlands; ^5^ Centre for Psychedelic Research, Division of Psychiatry, Imperial College London, London, England, United Kingdom; ^6^ Section for Clinical Addiction Research (RusForsk), Oslo University Hospital, Oslo, Norway; ^7^ Department of Psychology, University of Oslo, Oslo, Norway

**Keywords:** psychedelics, psilocybin, lysergic acid diethylamide (LSD), N,N-dimethyltryptamine (DMT), survey

## Abstract

**Background:**

Interest in the therapeutic effects of classical psychedelics has risen recently. However, little epidemiological knowledge exists about the use of classical psychedelics in Scandinavian countries. Additionally, there is a limited understanding of what factors drive self-reported improvement in wellbeing. The aim of this study was to investigate the relationship between the use of classical psychedelics and outcomes related to subjective wellbeing in an adult, Norwegian-speaking sample. We examined how contextual and phenomenological variables were associated with self-reported subjective wellbeing.

**Methods:**

Using an anonymous internet survey, we recruited Norwegian speaking subjects who have had a memorable experience after taking a classic psychedelic substance. Data are presented by using descriptive statistics about the sample and two hierarchical regression analyses. The first regression analysis examined contextual variables, and the second examined variables related to acute phenomena during the experience.

**Results:**

The survey showed that 85% of the sample reported a small to large positive change in subjective wellbeing after their experience with classical psychedelics. Integration, ego dissolution, and emotional breakthrough had a clear, positive predictive effect on the participants’ self-reported subjective wellbeing. Variables with lower but significant effects were the degree of challenging experiences, settings associated with nature or ceremony, and a therapeutic or seeking intention.

**Conclusion:**

The use of classical psychedelics leads to an increase in subjective wellbeing for the majority of the participants. This relationship seems dependent upon various experiential aspects of acute subjective drug effects. These findings should be viewed as hypothesis-generating rather than confirmatory due to the study’s limitations.

## Introduction

Numerous factors influence an individual’s response to psychedelic substances. These predictors encompass the individual’s personality, mindset, prior experiences with psychedelics, expectations, and social and environmental circumstances (Studerus et al., 2012). Mystical experiences have been shown to drive improvement in wellbeing following psychedelic use, while some studies suggest that other phenomena, such as psychological insights and emotional breakthroughs, may be as important (Kangaslampi, 2023). However, our understanding of how the interplay of these factors collectively impacts an individual’s psychedelic experience and subsequent outcomes remains incomplete, necessitating further research.

This study aimed to investigate how contextual factors (e.g., setting), and specific phenomena related to the psychedelic experience (e.g., emotional breakthroughs) influence measures of subjective wellbeing (SWB) following the use of a classic psychedelic substance in a recreational setting. SWB is a multi-dimensional construct encompassing both feeling good and functioning well (Keyes, 2006). At its broadest level, it can be defined as life satisfaction, with key components also including positive and negative affect (Deci and Ryan, 2008).

Classic psychedelic substances are typically defined as serotonin-receptor agonists such as synthetic lysergic acid diethylamide (LSD) and some naturally occurring substances. The most common examples are psilocybin from various mushroom species, mescaline from peyote and San Pedro cacti, and dimethyltryptamine (DMT) – the primary psychoactive ingredient in the plant-based brew ayahuasca. These substances exert their effect primarily through the stimulation of the serotonin (5HT) 2A receptor. Naturally occurring psychedelic substances have been used for thousands of years for spiritual, religious and medicinal purposes (Nichols, 2016).

Following the 1930s, there was a widespread interest in psychedelics in therapeutic and scientific settings, as well as politically and culturally (Nichols, 2016). However, in the 1960s, the research and clinical use of psychedelics were curtailed, among other reasons, by political tensions around drug use. In recent decades, there has been a resurgence of work on the therapeutic use of psychedelics (Haave and Pedersen, 2020).

Several new studies have shown promising results in diverse areas such as treatment of anxiety, depression, substance use and obsessive-compulsive disorder (OCD) (Andersen et al., 2020). The most recent trials are also assessing new indications such as state and trait anxiety (Holze et al., 2023), anorexia (Kelmendi et al., 2022) and body dysmorphic disorder (Schneier et al., 2023). We are not aware of any published studies on psilocybin or psilocybin-assisted psychotherapy as treatment for post-traumatic stress disorder (PTSD), however there is ongoing research actively exploring its potential (Choi et al., 2024; Khan et al., 2022). Although clinical trials are widespread, and do seem to deliver promising results, they apply strict selection criteria for participants to ensure safety. This leaves uncertainty about generalizability beyond specific diagnostic conditions and raises questions about the effects on users that would be excluded from clinical trials.

In the general population, an increase has been observed in recreational use of psychedelics, reaching numbers upwards of 20% increase in Great Britain (Winstock et al., 2021). In a representative sample of the Norwegian population, 8% reported having tried psilocybin (Jacobsen et al., 2021). However, little epidemiological knowledge exists about the use of classical psychedelics in Scandinavian countries. The apparent rise in recreational use of classic psychedelics, and the potential for clinical use as well, highlights the need for more knowledge in this area.

Psychedelic substances are known to induce both pleasurable and challenging experiences. Typical negative effects induced by psychedelics include anxiety, panic and paranoia (Johnson et al., 2008). Transient physiological symptoms such as dizziness, nausea and increased heart rate may occur (Johnson et al., 2008). Using the same data set as the current study, Kvam et al. found that 23.1% of the participants reported persisting adverse reactions after taking a psychedelic substance, the most frequent being sadness/dejection (3.5%), anxiety/nervousness (2.6%), and headache (1.8%) (Kvam et al., 2023). Carbonaro et al. investigated recreational psychedelic users who experienced psychologically challenging experiences (Carbonaro et al., 2016). Of the 2000 participants in the study, 11%described putting themselves or others in danger, 2.6% reported physical aggressive behaviour, and 2.7% sought medical help. Adverse effects of psychedelics, commonly referred to as “bad trips,” are particularly associated with high-dose use of psychedelic substances in a non-controlled setting characterized by inadequate preparation and psychological support (Roseman et al., 2017; Kopra et al., 2022; Carhart-Harris et al., 2018). According to the current understanding of the use of psychedelics, attention to contextual factors, administration form, and underlying health conditions are crucial to minimize the risk of adverse effects and risky behaviour (Schlag et al., 2022).

The ability of psychedelics to enhance neural and mental plasticity holds the potential for transformative effects, which are considered crucial for their therapeutic benefits (Carhart-Harris and Nutt, 2017). This increase in plasticity, when combined with psychotherapy, is believed to impact protective factors associated with long-term wellbeing, such as adaptation and resilience (Kocarova et al., 2021). A model that integrates psychedelic-induced plasticity within a supportive therapeutic context is proposed to have transdiagnostic potential (Kocarova et al., 2021).

As there is a recognized, ongoing negative relationship between wellbeing and mental difficulties (Lamers et al., 2015), prioritizing the safeguarding and enhancement of wellbeing can be considered pivotal (Mans et al., 2021). Additionally, initiatives and interventions are suggested not only for alleviating symptoms in clinical populations but also for individuals who already experience high levels of wellbeing. This approach aims to further enhance wellbeing and reduce the risk of mental difficulties (Sin and Lyubomirsky, 2009). Consequently, it is important to deepen our understanding of whether and how a psychedelic experience can influence wellbeing outside the therapeutic context as well.

Furthermore, it is imperative to study the correlation between various factors and long-lasting outcomes following a psychedelic experience, as well as how these factors interact with each other (St Arnaud and Sharpe, 2023). This understanding can contribute to a more profound comprehension of the necessary elements to optimize positive outcomes associated with the use of psychedelics. This becomes increasingly relevant as the recreational use of psychedelic substances remains prevalent in the Western world and is expected to continue growing based on current trends (Farah et al., 2024; Haijen et al., 2018; Winstock et al., 2017). Additionally, enhanced knowledge will be pivotal in the potential utilization of psychedelic substances for therapeutic purposes.

The present article offers an additional analysis of a survey previously presented by Kvam et al. (2023). The previous article described what characterized a memorable experience with a classic psychedelic drug, as well as the benefits and adverse reactions of such an experience in the short and long term, by using descriptive statistics. In contrast, the current article shifts its focus to analysing the factors that contribute to changes in subjective wellbeing.

The main objective of this study was to examine how predictors related to set and setting, as well as phenomena (i.e., ego dissolution, emotional breakthrough and challenging experiences) during a psychedelic experience, are associated with perceived changes in subjective wellbeing. We explored the following predictors: setting, type of intention, degree of intention, preparation, desire for change, integration, ego dissolution, emotional breakthrough, and challenging experiences.

The sub-aims of the study were 1) to study the degree of self-reported changes in subjective wellbeing after a psychedelic experience, 2) to study how the contextual predictors of preparation, setting, intention, desire for change and integration correlate to subsequent self-reported measures of subjective wellbeing, and 3) to study how the magnitude of rating of emotional breakthrough, challenging experiences and ego dissolution during a psychedelic experience correlate to subsequent self-reported measures of subjective wellbeing.

## Materials and methods

### Participants

Our cross-sectional study draws from responses gathered from anonymous Norwegian adult participants. They completed a 119-item internet survey concerning the recreational use of classic psychedelic substances. The criteria for participation were 1) being 18 years or older, 2) being able to read, write, and speak fluent Norwegian, and 3) having had a memorable experience after taking a psychedelic substance. The survey asked for the following demographic information: age, gender, education level, annual income, and marital status. See Kvam et al., (2023) for recruitment, participation criteria, survey items, and respondent characteristics, as well as ethical considerations and data protection (Jacobsen et al., 2021). Due to the sensitive nature of the data and conditions in the consent, we make the data set available on reasonable request.

### Measures

After reading project information and providing demographic information, participants proceeded to answer several questions regarding their experience. Among them were questions related to the following topics relevant to this study.

*Setting* referred to the participant’s location during the psychedelic experience, with response options referring to physical surroundings and circumstances. *Intention Type* was a measure of what the participant considered the main reason for taking the psychedelic substance. *Preparation* rated to what extent they would say they were prepared for what the psychedelic experience could entail beforehand. The response options for this, and the next three variables, were ranked on a five-point Likert scale from “To a very small extent” to “To a very large extent.” *Intention Degree* was a measure of the extent to which the participant evaluated having entered the psychedelic experience with a clear intention. *Desire for change* referred to the degree to which the participant evaluated having entered the psychedelic experience with a desire for change. *Integration* rated to what extent they had worked to transfer any experiences, thoughts, attitudes, and insights from the psychedelic experience to everyday life.

Additionally, we used total scores from the following established and standardized questionnaires, which have been translated into Norwegian and examined for psychometric properties (Andersen and Holmøy, 2025; Andersen et al., 2025), as reported below.

#### Emotional Breakthrough Inventory (EBI)

The questionnaire aims to assess a possible emotional breakthrough (Roseman et al., 2019). EBI consists of six items that are rated on a visual analog scale from 0 (“No, not more than usual”) to 100 (“Yes, to a very large extent”). Examples of statements used in the questionnaire include “I faced difficult emotions that I usually avoid”. McDonald’s omega for EBI total in the current data set is 0.90.

#### Ego dissolution inventory (EDI)

The questionnaire seeks to assess a possible dissolution of the ego or self (Nour et al., 2016). The questionnaire has eight items rated the same way as EBI. Examples of statements used in the questionnaire include “All sense of self and identity dissolved.” McDonald’s omega for CEQ total in the current data set is 0.86.

#### Challenging experience questionnaire (CEQ)

The questionnaire is intended to assess challenging experiences under the influence of a psychedelic substance (Carbonaro et al., 2016; Barrett et al., 2016). The questionnaire consists of 26 items that are divided into the following subscales: fear, grief, physical discomfort, feeling of madness, isolation, death, and paranoia. The participant is asked to rate to what extent they experienced various phenomena associated with challenging experiences. Examples of such phenomena include “Isolation and loneliness,” and “Feeling that others were out to get me.” The phenomena are rated on a six-point Likert scale from “None; not at all” to “Extremely, more than ever before in my life.” McDonald’s omega for CEQ total in the current data set is 0.94.

We constructed the Subjective Wellbeing dependent variable (SWB) as a sum score based on ten items from the survey covering short-term and persisting effects of the experience. Although not a standardized questionnaire for subjective wellbeing, it was considered an appropriate measure for this study. The questions were related to sustained changes in wellbeing/satisfaction, purpose in life, meaning in life, relational changes to other people, attitudes towards life, relationship to nature, mood/affect, behaviour, spirituality, and attitudes towards death. Responses to the relevant items were ranked on a seven-point Likert scale from “Significant positive change that I consider desirable” (value 7) to “Significant negative change that I consider undesirable” (value 1). The possible range of the SWB variable was 10–70. The midpoint is 45, reflecting a neutral answer (no change) on average on all ten items. McDonald’s omega for the constructed SWB measure was 0.92.

### Data analysis

The variable Setting consisted of seven dummy-coded variables with the reference category “at home.” The variable Intention Type consisted of three dummy-coded variables with the reference category “recreational intention.” The dummy variables were compared to the reference category to see if they had larger or smaller effects, but it was not indicative of their magnitude.

We conducted two hierarchical regression analyses. The first one examined predictors related to set and setting, while the second looked at predictors associated with phenomena during the experience and their association with sustained changes in subjective wellbeing.

We organized the order from the lowest expected effect to the highest expected effect to see how much the assumed most important predictors explain when accounting for several variables simultaneously. Unfortunately, as there are few studies that shed light on the importance of these factors relative to each other, the order was largely based on our judgment of the highlighted factors in the research field. It is important to remember that the effect size of the steps is affected by their position in the order. Later steps will have a lower effect size as more variables are accounted for simultaneously.

The first hierarchical regression analysis was conducted in multiple steps, with additional predictor variables being added at each step to see how they contributed to the explained variance in subjective wellbeing. Age and gender were added in the first step, the setting in the second, type of intention in the third, preparation level, degree of intention, and desire for change in the fourth, and finally integration in the fifth and last step. The second hierarchical regression analysis had an identical first step to the first analysis (age and gender), but proceeded with CEQ, EBI, and EDI sum scores in subsequent steps. To keep the models simple, we opted not to include interaction effects, as these can be difficult to interpret.

After conducting the regression analyses, we examined variance distribution, normality, and linearity. We used histograms, scatterplots, and PP-plots from the final step of the regression analysis. Bivariate relationships between all variables were examined using Pearson’s *r*, and the assumption of multicollinearity was assessed by reviewing the correlation matrix, variance inflation factor test (VIF), and tolerance scores for the variables. All statistical analyses were performed using SPSS statistics version 25.

## Results

In total, 841 participants completed the survey. We removed 71 participants who did not meet the inclusion criteria or had low variance or low response time. This left us with a sample of 770 participants. For a full report of the participant demographics and other descriptives, see Kvam et al., (2023).

### Descriptives

The mean SWB score was *M* = 56.2 (*SD* = 8.79). The variable showed a clear left-skewed distribution (skewness = −0.498, kurtosis = 0.181). An internal consistency calculation yielded a Cronbach’s alpha of 0.93, which indicates a very high consistency. Only 9%, or 72 participants, fell below the midpoint at 45, indicating that they had experienced negative changes. Six percent, or 44 participants, had a score of 44–46, indicating no changes in subjective wellbeing after their experience. A total of 85%, or 654 participants, had experienced a small to large positive sustained change in subjective wellbeing following their experience with a psychedelic substance. The mean score of 56 also indicated that many participants had relatively noticeable positive changes in the outcome variable of subjective wellbeing. The mean sum score for EBI was *M* = 338.1 (*SD* = 161.64, maximum score is 600), mean of mean score was *M* = 56.36 (*SD* = 26.99). For EDI the sum score was *M* = 460.2 (*SD* = 199.76, maximum score 800), and the mean of mean scores *M* = 57.52 (*SD* = 24.96). CEQ had a clear right-skewed distribution where most participants reported a low degree of challenging experiences (sum score *M* = 52.3, *SD* = 24.12, maximum score 151, mean score *M* = 2.01, *SD* = 0.93).

### Regression analyses

In the first analysis (Table 1), age was initially a significant variable in the first and second step, but not in later steps. In the second step, Nature and Ceremonial settings remained significant in the final model, whereas Other and Urban turned nonsignificant. Therapeutic and Seeking intentions remained significant in the last model, whereas none of the fourth step variables remained significant. Integration had a significant effect in the last step. In summary, Nature and Ceremonial setting, Therapeutic and Seeking intentions, and Integration prevailed as significant predictors of subjective wellbeing. Explained variance for the final model was *R* ^*2*^ = 0.39 (Δ*F*[1, 752] = 218.58).

**TABLE 1:** Unstandardized regression coefficients from hierarchical regression analyses in five steps with wellbeing as dependent variable.

Variable	Step 1	Step 2	Step 3	Step 4	Step 5
	*b*	*b*	*b*	*b*	*b*
(Constant)	54.040	54.466	51.798	45.960	35.036
Age	0.958**	0.652*	0.398	0.471	0.463
Sex (*0 = Woman*)					
Other	−3.533	−3.822	−4.322	−4.411	−4.690
Man	0.091	0.274	0.154	0.102	0.731
Setting (*0 = At home*)					
Other		−1.613	−0.453	−0.218	1.163
Nature		2.315**	2.555**	2.623***	2.893***
Urban		−3.872*	−1.620	−1.562	0.506
Ceremonial		7.448***	5.337*	4.542**	4.256**
Festival		−1.825	−0.701	0.226	1.209
Therapy		4.071*	1.704	1.197	0.451
Other		−3.361**	−2.240*	−1.698	−0.707
Intention (*0 = recreational*)					
Therapeutic			5.960***	4.111***	2.580***
Seeking			5.488***	4.303***	2.210*
Escape			−1.236	−0.785	−1.030
Preparation				0.611*	0.427
Intention				0.083	0.096
Need for change				1.227***	0.501
Integration					3.860***
*R* ^2^	0.015**	0.086***	0.189***	0.215**	0.392***
Δ *R* ^2^	—	0.071***	0.103***	0.026***	0.177***
FChange	F(3,766) = 3.939	F(7,759) = 8.387	F(3,756) = 32.147	F(3,753) = 8.225	F(1,752) = 218.575

*Note*. N = 770. *p < .05, **p < .01, ***p < .001.

In the second analysis, age remained significant in the final step, as did all three standardized measures as significant predictor variables for subjective wellbeing (Table 2). The effect of CEQ changed from negative to positive when EDI and EBI were included, suggesting a potential interaction between CEQ and the degree of EDI and EBI. Explained variance in the final model was *R* ^2^ = 0.44 (Δ*F*[1, 763] = 163.54).

**TABLE 2:** Unstandardized regression coefficients from hierarchical regression analysis in four steps with subjective wellbeing as dependent variable.

	Step 1	Step 2	Step 3	Step 4
	*b*	*b*	*b*	*b*
(Constant)	54.040	56.474	47.933	42.552
Age	0.958**	0.912**	0.822**	0.904**
Sex (*0 = Woman*)				
Other	−3.533	−3.135	−3.018	−4.445
Man	0.091	0.186	0.703	0.889
CEQ		−0.046**	−0.076**	0.075**
EBI			0.029**	0.022**
EDI				0.017**
*R* ^2^	0.015**	0.031***	0.316***	0.437***
Δ *R* ^2^	—	0.016***	0.285***	0.121***
F change	F(3,766) = 3.939	F(1,765) = 12.519	F(1,764) = 318.024	F(1,763) = 163.543

Note. *N* = 770. *p < .05, **p < .01, ***p < .001.

## Discussion

The aim of the study was to examine how contextual factors and various phenomena during a psychedelic experience were associated with subjective wellbeing (SWB). The sub-aims were to study the degree of self-reported changes in SWB following a psychedelic experience, how the contextual predictors preparation, setting, intention, desire for change and integration correlate to self-reported measures of SWB, and to study how the magnitude of rating of emotional breakthrough, challenging experiences and ego dissolution during a psychedelic experience correlate to self-reported measures of SWB.

### Degree of self-reported changes in subjective wellbeing

The first sub-aim was to study the degree of self-reported changes in SWB following a psychedelic experience. Our study suggests that recreational use of psychedelics in a Norwegian convenience sample is associated with increased subjective wellbeing for most of the participants (85%). A large majority experienced smaller to larger degrees of positive change in subjective wellbeing. This is consistent with previous studies (Hendricks et al., 2015; Johansen and Krebs, 2015; Krebs and Johansen, 2013). The findings are intriguing as they suggest that psychedelics have therapeutic potential beyond the clinical context. Importantly, none of the predictors (preparation, setting, intention, desire for change, integration, emotional breakthrough, ego-dissolution, challenging experiences) were found to be associated with negative changes in subjective wellbeing. The differences between the predictors primarily revolve around the extent of positive changes reported by the participants.

### The contextual predictors

The second sub-aim was to study how the contextual predictors preparation, setting, intention, desire for change and integration correlate to self-reported measures of subjective wellbeing. Our results indicate that the degree of integration appeared to have the most significant effect on wellbeing, as it contributed to the largest proportion of explained variance in the first analysis and reduced the effects of other predictors. The other predictors which remained statistically significant were nature, ceremony, therapeutic intention and seeking intention.

In our study integration appears to have the strongest effect on subjective wellbeing. This may point to the essential role that integration plays in the recovery and maximization of potential benefits from a psychedelic experience (Breeksema et al., 2020). The findings are consistent with St Arnaud and Sharpe (2023), where integration had the strongest association with aspects of wellbeing. Our results also support the relevance of integration work in a clinical setting (Richards, 2017; Grunder et al., 2023). The findings highlight the complexity of psychedelic substances and their therapeutic potential. While these substances can facilitate new insights and perspectives, the individual must subsequently understand and implement relevant changes in emotions, thoughts, and behaviors. The central role of integration may suggest that changes can be enhanced or made more durable with integration, and that the participant must actively make use of the increased plasticity introduced by the substances. This aligns with the current understanding that neuroplasticity is not inherently positive. To speculate on the observed effect of integration on wellbeing, factors such as the time and space available during the integration phase, and the level of support from peers, community and therapists, may all play significant roles (Grunder et al., 2023). Our study did not consider whether participants integrated their experiences alone or with others. However, we can speculate on the importance of social and emotional support after a psychedelic experience. Without adequate support, participants may feel alienated and vulnerable after a psychedelic experience, and the integration process becomes relevant in countering such an experience (Watts et al., 2017). Integration work is likely particularly relevant in the context of challenging experiences or re-experiencing painful memories, as a temporary increase in symptomatology may occur. Safe and supportive integration work can be important in guiding the participant through this phase, and in incorporating the difficulties into the participant’s therapeutic process (Gorman et al., 2021). As with other forms of psychotherapy, the therapeutic alliance is a central element. The alliance is important for the experience of safety, and for the person to feel comfortable enough to engage with and attempt to overcome intense emotional experiences (Breeksema et al., 2020). Thus, both preparation and integration are crucially important.

In line with previous studies that present a positive association between aspects of group setting and subsequent wellbeing (St Arnaud and Sharpe, 2023), our results highlight the connection between a ceremonial setting and subjective wellbeing. This strengthens the assumption that a perceived safe, comfortable, and well-regulated setting is beneficial for both the acute experience and long-term outcomes (Johnson et al., 2008; Haijen et al., 2018; Capaldi CAP et al., 2015).

Our findings indicate a positive effect of taking psychedelics in nature. This resonates with previous studies that highlight the beneficial effects of feeling comfortable in the environment and experiencing a sense of belonging and connection to nature (Capaldi CAP et al., 2015; Kettner et al., 2019; Kettner et al., 2021). Our results imply that being in a therapeutic setting is positively associated with subjective wellbeing, but the effect does not remain significant when accounting for the type of intention and integration. These findings are somewhat unexpected, as previous research has shown a positive association between a therapeutic setting and beneficial phenomena during the psychedelic experience, which in turn is related to wellbeing (Roseman et al., 2017; Carhart-Harris et al., 2018; Reiff et al., 2020). It is possible that the effect would have been stronger if the outcome measure was focused on acute effects. However, our findings are consistent with Haijen et al. (2018), which showed that a therapeutic setting did not have a significant influence on psychedelic experiences. Being comfortable and feeling safe in the environment may be more relevant than whether the surroundings are specifically perceived as therapeutic. At the same time, our results may be influenced by the subjective and varying definition of a therapeutic setting. In the present study, therapeutic intention was defined as a desire for new insights, increased quality of life, symptom reduction, and processing of difficult memories or emotions. The results are consistent with previous findings from recreational psychedelic use, where intentions related to growth and self-insight are associated with higher scores of wellbeing (St Arnaud and Sharpe, 2023; Haijen et al., 2018; Reiff et al., 2020). A somewhat surprising finding is that we did not find a significant association between the predictor of intention degree and subjective wellbeing. It is possible that the type of intention is more relevant than the intensity of intention, or that much of the effect of intention intensity disappears when accounting for preparation level or desire for change.

The findings showing a positive correlation between a seeking intention and subjective wellbeing are in line with previous research by Haijen et al. (2018), which showed that using psychedelic substances with the intention of spiritual connection has a positive impact on wellbeing. At the same time, our findings indicate that spirituality is considered an aspect of wellbeing that increases after a psychedelic experience (Mans et al., 2021). The results can also be interpreted in line with the assumption that spiritual intention facilitates ego-dissolution (Haijen et al., 2018; Russ et al., 2019). In addition, the association between spirituality and wellbeing could be attributed to the correlation between having a spiritual intention and lifetime use of psychedelic substances (Haijen et al., 2018). The correlation indicates that individuals who have taken psychedelic substances multiple times are more likely to enter psychedelic experiences with the intention of having a spiritual connection and subsequently report higher levels of wellbeing. It is possible that there is a similar tendency in our sample, as the majority of participants reported having used psychedelic substances several times in the past. Caution should therefore be exercised when generalizing the results, as the sample might consist of participants who already score high on spirituality even before the psychedelic experience. Therefore, it becomes difficult to interpret the isolated effect of seeking intention.

### The magnitude of rating of ego-dissolution, emotional breakthrough and challenging experiences

The third sub-aim was to study how the magnitude of emotional breakthrough, challenging experiences and ego dissolution during a psychedelic experience correlate to subsequent self-reported measures of SWB. In our second regression analysis, high levels of ego-dissolution and emotional breakthrough were found to be associated with positive changes in subjective wellbeing, while challenging experiences showed a weak positive association in the final step of the analysis.

The results from the second regression analysis show that a high degree of challenging experiences is positively associated with subjective wellbeing when linked to ego-dissolution and emotional breakthrough. However, when looking at challenging experiences alone, there is a negative association. This is consistent with the assumption that challenging experiences can be advantageous if the participant experiences an emotional breakthrough (Carbonaro et al., 2016; Haijen et al., 2018; Barrett et al., 2016). The results suggest that the effect of challenging experiences may be related to the experience of psychological insight or emotional breakthrough. This is in accord with previous studies that suggest challenging experiences are not always negative and can occasion emotional breakthrough (Palmer and Maynard, 2022; Gashi et al., 2020). By accounting for emotional breakthrough, our results show that challenging experiences are not necessarily a negative phenomenon nor predictor. Our findings suggest that the confrontation with challenging emotions can be an important component of a potential therapeutic process, leading to emotional release or insight (Nutt et al., 2020). The results can be seen as part of a growing body of research that indicates that the effect of challenging experiences is part of a complex interplay with other factors and phenomena (Carbonaro et al., 2016; Barrett et al., 2016). However, it should be noted that the sample consistently scored low on challenging experiences and may not be representative of the effect of challenging experiences on outcome measures. It is possible that participants may have consciously omitted potentially more challenging experiences, as they were instructed to select only one memorable experience, possibly prioritizing positive ones.

The results indicate that emotional breakthrough is associated with positive changes in subjective wellbeing. These findings are consistent with previous psychedelic studies (Roseman et al., 2019; Palmer and Maynard, 2022; Nutt et al., 2020; Nygart et al., 2022). It is particularly interesting that the results show that emotional breakthrough explains such a large proportion of the variation in subjective wellbeing. Thus, our results support the importance of emotional breakthrough, which is considered an important process factor underlying positive psychotherapy outcome in general (Sonderland et al., 2023). As several predictors in this study, emotional breakthrough likely interacts with other factors. For example, a therapeutic context has previously been shown to predict a stronger degree of emotional breakthrough (Roseman et al., 2019). An interesting aspect of our findings is that participants reported a high degree of emotional breakthrough even though most of the sample did not consume the substances in a therapeutic context. Nevertheless, it is possible that having a therapeutic intention may have influenced the occurrence of emotional breakthrough phenomena. Emotional breakthroughs are often related to personal and interpersonal insight, and the acquired insight may become clearer in the period following the psychedelic experience (Roseman et al., 2019; Gashi et al., 2020). Therefore, integration can be considered another relevant factor in this context, and the effect of emotional breakthrough might have been affected if the study included integration in the second regression analysis.

Our results indicate that ego dissolution is a positive, significant predictor of subjective wellbeing. This is consistent with previous findings where ego dissolution is associated with beneficial subjective changes (Kettner et al., 2019; Lebedev et al., 2016; Smigielski et al., 2019; Uthaug et al., 2019; Uthaug et al., 2018; Amada et al., 2020). However, like the other phenomena in this study, it is difficult to pinpoint exactly what makes ego dissolution have a beneficial effect on subjective wellbeing. One way to interpret the findings is to examine the external perspective that one can gain on oneself and one’s life (Amada et al., 2020; Agin-Liebes et al., 2021). It seems that the experience of ego dissolution mitigates the focus on oneself, and one’s concerns, creating the opportunity to see beyond the confines of habitual thoughts, feelings and behaviours. This can be supported by the mediating effect that ego dissolution has been shown to have on self-awareness during psychedelic use (Amada et al., 2020). In this context, biological mechanisms also become relevant, as it has been suggested that more interconnected brain activity is correlated to the subjective experience of unity often associated with ego dissolution (Russ et al., 2019; Tagliazucchi et al., 2016). Another complementary interpretation is that ego-dissolution facilitates psychological insight (Kałużna et al., 2022), potentially contingent on integration work following the psychedelic experience. For example, an experience of ego-dissolution in safe and regulated circumstances can be therapeutically effective, facilitating insight, new perspectives, and self-actualization (Lebedev et al., 2016). Thus, ego-dissolution, as the previously mentioned phenomena, is likely to covary with other factors.

### Limitations

While the results of this study suggest a positive effect of psychedelic substances on subjective wellbeing, it is important to interpret the findings with several limitations in mind. First of all, the study was not preregistered as it is based on a student thesis, and it should therefore be regarded as an exploratory study. Furthermore, the results may be influenced by the fact that the sample appears to have generally positive attitudes towards psychedelics, as 95% of participants agreed or strongly agreed that psychedelic substances should be used therapeutically. This skewness in the sample could be due to the study’s recruitment through internet and social media advertisement. Additionally, the study may have attracted participants with previous positive psychedelic experiences, whereas others with unfavourable experiences may have been more hesitant to share their experiences.

The cross-sectional design cannot confirm a causal relationship between psychedelic use and subjective wellbeing. It is possible that other factors not accounted for in this study, such as personality traits or life experiences, could affect both psychedelic use and subjective wellbeing. As many of the participants reported an experience from the past, difficulties in accurately remembering the experience or comparing their wellbeing before and after the experience might have affected the responses. Additionally, several participants left an anonymous comment in the survey regarding the challenge of choosing only one experience to highlight, as they had multiple psychedelic experiences that were equally significant. These participants reported that the overall sum of their experiences was more important than any individual experience, thus providing an incomplete picture of their experiences with psychedelics when asked to highlight only one. It is reasonable to assume that this applies to several participants with multiple experiences, which reduces the representativeness of the responses. The self-reported data can also be susceptible to response bias as some participants may have provided socially desirable answers influenced by implicit or explicit expectations from research literature, media, peers or other factors.

The study has several limitations related to the measurements. The outcome variable lacked a standardized measure of subjective wellbeing, although the included questions attempted to capture its central aspects. The setting measure only compared alternatives without assessing their quality. The integration measure could have been more specific to go deeper into participants’ integration processes post-experience. While the EDI precisely measures ego-dissolution, it overlooks other aspects of mystical experiences captured by the MEQ30. Many studies use the MEQ30 to measure mystical experiences with subcategories such as ineffability, transcendence of time and space, and the experience of unity (Andreas Aamodt Andersen et al., 2024). Choosing the EDI over the MEQ30 was a trade-off made during the design of the study to limit the total number of items in the questionnaire. The abstract nature of the EBI and its assumption about participants’ affective awareness may limit its applicability. While combining CEQ, EBI, and EDI in regression analysis is beneficial, the study lacks information on the interaction between predictors and other factors, despite the hierarchical research design indicating such interactions occur.

## Conclusion

In a Norwegian-speaking sample, 85% of recreational classical psychedelic users experienced small to large positive changes in wellbeing. Integration, ego-dissolution, and emotional breakthrough were important predictors for subjective wellbeing, while degree of challenging experiences, nature and ceremony settings, and therapeutic and seeking intention had lower but significant effects. The results should be viewed as hypothesis-generating rather than conclusive. More epidemiologic data is needed to map a more complete population of psychedelic users and creative recruitment strategies are needed to include underrepresented subgroups in future research. More detailed knowledge is needed about specific predictors, their effective elements and interactions with other factors affecting positive and negative outcomes after psychedelic use.

## Data Availability

Dataset may be obtained on reasonable request. Requests to access these datasets should be directed to CG, cato.gronnerod@psykomatikk.no.

## Abbreviations

LSD, lysergic acid diethylamide; DMT, N,N-dimethyltryptamine; LTI, life-threatening illness; AUD, alcohol use disorder; HPPD, hallucinogen persisting perception disorder; 5-MeO-DMT, 5-methoxy-N,N-dimethyltryptamine; PTSD, post-traumatic stress disorder; OCD, obsessive-compulsive disorder; MDMA, methylenedioxymethamphetamine; EDI, Ego-Dissolution Inventory; EBI, Emotional Breakthrough Inventory; CEQ, Challenging Experience Questionnaire; ADHD, attention deficit hyperactivity disorder; ASD, autism spectrum disorder.
